# Role of E5 from HPV16 in the Evasion of the Immune Response

**DOI:** 10.3390/ijms27041985

**Published:** 2026-02-19

**Authors:** Aislinn C. Pérez-Morales, Minerva Maldonado-Gama, Marisela Méndez-Armenta, Fernando Esquivel-Guadarrama, Lourdes Gutierrez-Xicotencatl

**Affiliations:** 1 Centro de Investigación Sobre Enfermedades Infecciosas, Instituto Nacional de Salud Pública, Cuernavaca 62100, Mexico; acpmiaw@gmail.com (A.C.P.-M.); mmaldona@insp.mx (M.M.-G.); 2 Centro de Investigación en Dinámica Celular, Universidad Autónoma del Estado de Morelos, Cuernavaca 62210, Mexico; 3 Departamento de Neuroquímica, Instituto Nacional de Neurología y Neurocirugía, “Manuel Velasco Suárez”, Ciudad de México 14269, Mexico; mmendezarmenta@hotmail.com; 4 Facultad de Medicina, Universidad Autónoma del Estado de Morelos, Cuernavaca 62350, Mexico

**Keywords:** E5 oncoprotein, immune evasion, interferon signaling, TGF-β pathway, MHC-I and II

## Abstract

Human papillomavirus type 16 (HPV16) persistence relies on early viral mechanisms that synchronize oncogenic signaling with immune evasion, with the E5 oncoprotein serving as a central regulator of the viral cycle and the initiation of cell transformation. This review integrates current evidence on how E5 reconfigures host cell dynamics: first, by hijacking signaling pathways such as EGFR, MAPK/ERK, and PI3K/AKT to drive keratinocyte proliferation and survival; and second, by organizing a multi-step immune evasion strategy. We detail how E5 suppresses innate antiviral responses, specifically by repressing IFN-κ and IFN-β via interference with IRF1, TGF-β/SMAD, STING, and MAVS signaling. Simultaneously, E5 interferes with the adaptive immunity by disrupting MHC-I trafficking and impairing MHC-II maturation. Furthermore, preclinical studies utilizing various vaccine platforms targeting HPV16 E5 have demonstrated the capacity to reduce tumor burden and significantly increase survival rates. By integrating these molecular and immunological checkpoints, we highlight the role of E5 in sustaining viral persistence and underscore its potential as a high-value target for next-generation immunotherapeutic and vaccine-based strategies.

## 1. Introduction

High-risk (HR) human papillomaviruses (HPVs) are the primary cause of several cancers, particularly cervical cancer [1]. HPVs are double-stranded DNA viruses with tropism for mucosal and stratified epithelial tissues [1]. While most HPV infections are cleared within approximately two years, HR-HPV types can persist and progress to premalignant lesions that may eventually develop into invasive cancer [2].

Infection initially occurs in basal epithelial cells, but early viral replication is non-productive because the viral DNA remains episomal. As keratinocytes differentiate, viral genome replication is activated, and non-structural viral proteins are expressed. In the upper layers of the epithelium, during terminal differentiation, structural capsid proteins are synthesized and assembled into virions, which are then released through the basolateral surface of keratinocytes [3]. However, during persistent infection, dysregulation of the cell cycle promotes proliferation without differentiation, ultimately favoring cellular transformation. The viral oncogenes E5, E6, and E7 are central to this process, each contributing to immune evasion, genomic instability, and cancer progression [4].

Keratinocytes possess a broad repertoire of innate immune sensors that detect viral components and trigger cytokine production, which is essential for maintaining local immune surveillance [5]. These epithelial cells are the primary targets of HPV infection. The E5 oncoprotein has been shown to suppress Interferon-kappa (IFN-κ) expression, downregulate interferon-stimulated genes (ISGs), and interfere with viral genome integration [6]. Moreover, E5 reduces the expression of key immunomodulatory molecules such as transforming growth factor beta receptor II (TGF-βRII), thereby attenuating TGF-β signaling and promoting carcinogenic processes [6].

This review summarizes the cellular targets and molecular mechanisms through which the E5 oncoprotein modulates host pathways to evade immune detection and facilitate HPV persistence. Furthermore, we discuss the role of E5 as a potential target for prophylactic and therapeutic strategies under development to counteract E5-mediated immune evasion.

## 2. Methodology

This article employs a narrative review methodology to provide a comprehensive qualitative synthesis of current knowledge on the role of HPV16 E5 oncoprotein in modulating the host immune response. A comprehensive literature search was conducted across the PubMed, Scopus, and Web of Science databases using keywords including “HPV E5”, “immune evasion”, “innate immune response”, “adaptive immune response”, “interferon signaling”, “TGF-β pathway”, “MHC-I and MHC-II”, and “antigen presentation”.

The selection of sources prioritized seminal mechanistic studies and recent peer-reviewed research that describes the molecular interactions of E5 in human epithelial models. Data were synthesized using a thematic analysis approach, categorizing E5’s activities into four different axes: (1) E5 in cell transformation, (2) innate immune response, (3) adaptive immune response, and (4) E5 as a therapeutic target. This review focuses on the synergy between E5-mediated signaling disruption and the evasion of innate and adaptive immune surveillance.

## 3. Role of E5 from HPV16 in Cell Transformation

The HPV16 E5 oncoprotein is a highly hydrophobic 83 amino acid protein characterized by three transmembrane α-helices (residues 8–30, 37–52, and 58–76) [7]. By localizing to various cellular compartments, including the endoplasmic reticulum (ER), the Golgi apparatus (GA), the plasma membrane, and early endosomes, E5 exerts diverse functional effects in keratinocytes, initiating cellular transformation [7].

During productive infection, E5 is involved in viral genome amplification and activation of late gene expression [8]. Additionally, HPV16 E5 contributes to cell proliferation and regulates signal transduction pathways in infected cells [9]. It is expressed in cervical intraepithelial neoplasia (CIN) 1 or 2, favoring immortalization of human keratocytes in conjunction with E6 and E7 oncogenes, suggesting that E5 plays a vital role in the initial stages of cervical cancer (CC) [10].

E5 can bind the 16-kDa subunit C of the V-H+-ATPase, thereby preventing the acidification required for lysosomal protein digestion, which increases epidermal growth factor receptor (EGFR) recycling [8]. At the same time, E5 interacts with c-Cbl, an E3 ubiquitin ligase, thereby preventing ubiquitination of the EGFR for degradation [11]. These events induce constitutive signaling, in which EGFR activation drives the cell into a permanent state of mitogenesis. To sustain the transformed state, the E5 protein evades apoptosis by modulating both intrinsic and extrinsic signaling pathways [12]. It suppresses the intrinsic pathway by triggering ubiquitin-dependent degradation of anti-apoptotic Bax, a process mediated by PI3K-AKT signaling, as well as other pathways such as the cyclooxygenase-2 (COX-2), prostaglandin E2 (PGE_2_), and the cyclic adenosine monophosphate-dependent protein kinase (PKA) [13]. Additionally, E5 disrupts extrinsic apoptosis by downregulating CD95 (Fas) surface expression and inhibiting TNF-related apoptosis-inducing ligand (TRAIL) signaling [14]. Therefore, HPV16 E5 is a critical driver of early cellular transformation by uncoupling growth factor receptors from their natural degradation pathways and abolishing a multilayered barrier to apoptosis.

Regulation of inflammation by E5 is another mechanism by which this oncoprotein drives transformation. In healthy epithelium, p63 is expressed in proliferative, undifferentiated cells; as cells differentiate from the basal layer to the surface, p63 expression is negatively regulated by miR-203 in the upper epithelial layers. At the same time, miR-203 also interacts and reduces Suppressor of Cytokine Signaling 3 (SOCS3) expression, which is involved in inflammation. In this context, repression of miR-203 by HPV16 E5 maintains high p63 levels, forcing the cells into a proliferative loop and increasing SOCS3 levels, thereby decreasing inflammation [11]. These two events are crucial for the accumulation of genetic mutations that lead to cancer, and decreasing inflammation ensures that proliferative transformed cells are invisible to the immune system during the early stages of malignancy. Another inflammation pathway regulated by E5 is the modulation of miR-146a, which targets interleukin-1 receptor-associated kinase (IRAK1) and TNF receptor-associated factor 6 (TRAF6), thereby inhibiting NF-κB signaling and ultimately reducing inflammation, thereby facilitating immune evasion [15,16].

E5 HPV16 also drives cellular transformation through immune evasion by modulating antigen presentation, lipid raft remodeling, and suppressing the innate-adaptive bridge. In the case of the MHC-I (Major Histocompatibility Complex type I) present in the plasma membrane, E5 disrupts the surface expression of these molecules in cervical keratinocytes (W12 cells) by binding to the MHC-I heavy chain and retaining it in the Golgi apparatus [17]. The molecular hijacking is mediated by the di-leucine repeats in the first E5 N-terminal hydrophobic domain. In human keratinocytes and epithelial models (HaCat and HEK-293T cells), this same domain subverts the chaperone proteins BAP31 and calnexin, which effectively block MHC-I transport before it reaches the plasma membrane [18]. Consequently, by dismantling the antigen presentation machinery, E5 prevents CD8+ T cell activation, allowing the transforming cell to proliferate undetected [17]. Another mechanism by which E5 suppresses CD8+ T cell activity is by increasing caveolin-1 and ganglioside-1 (GM-1) levels within lipid rafts. This upregulation promotes the formation of caveolin/GM-1 complexes, a mechanism observed in various cervical cancer cell lines. These complexes exert a dual effect: suppressing CD8+ T lymphocyte activity while simultaneously amplifying EGFR-mediated proliferative signaling [19]. Finally, E5 impairs CD1d trafficking to the cell surface by destabilizing its interaction with calnexin. Because CD1d is required for NKT cell activation, this mechanism effectively prevents NKT cell recognition and inhibits the resulting cytokine response in cervical cancer cells [20].

## 4. Role of E5 in Innate Immune Response Evasion

The innate immune response plays a crucial role in controlling viral replication and propagation. Viruses encode proteins that specifically downregulate different mechanisms of the innate immune response. HPV is no exception: its oncoprotein E5, independently of its cell-transforming properties, also plays a vital role in suppressing the innate immune response, including interferon signaling pathways.

Interferons (IFNs) are a family of low-molecular-weight molecules with potent antiviral functions, induced by the cellular detection of pathogen-associated molecular patterns (PAMPs) through pattern recognition receptors (PRRs) [21]. The IFN family comprises three types of proteins (I, II, and III). Type I includes IFNα, −β, −κ, −ε, and −ω, type II includes IFNγ, and type III includes IFNλ. IFNs type I and III are mainly secreted in the innate immune response, and IFN II by the adaptive immune response [22]. Understanding the mechanisms by which E5 from HPV regulates IFN production and signaling is essential for developing future therapeutic strategies against this infection.

### 4.1. Inhibition of IFN-κ Production

In stratified healthy epithelia, keratinocytes constitutively express IFN-κ, but during infection, IFN-α and IFN-β secretion are stimulated, generating an antiviral response promoted by the upregulation of ISGs [23].

The primary canonical pathway for IFN signaling is the JAK/STAT pathway, but the mitogen-activated protein kinase (MAPK) and PI3K-AKT-mTOR pathways act as non-canonical pathways in regulating IFN-I, including IFN-κ in epithelial cells [24]. Specifically, in keratinocytes, the MEK/ERK pathway has a regulatory role in IFN-κ expression [25,26]. This regulation occurs through direct mechanisms, such as the activation of specific transcription factors that bind to the IFN-κ promoter, and indirect mechanisms, involving the functional repression of key immune regulators such as Interferon Regulatory Factor 1 (IRF1) and Signal Transducer and Activator of Transcription 1 (STAT1). Sustained MEK/ERK activation lowers IRF1 transcription activity and limits STAT1 nuclear accumulation, thereby reducing IFN-κ induction [27].

This control of IFN-κ expression becomes particularly relevant in the context of HPV16 infection, where the E5 oncoprotein acts as a key immunosuppressive regulator [28]. Recent studies have shown that E5 actively and specifically suppresses basal IFN-κ expression in infected keratinocytes, whereas it has little or no effect on the expression of other type I interferons such as IFN-α or IFN-β [6]. This suppression occurs even in the absence of exogenous stimuli and can be reversed by deleting E5 from the viral genome, demonstrating its role as a basal transcriptional repressor of IFN-κ [6]. In this context, the HPV16 E5 oncoprotein emerges as a central regulator of IFN-κ pathway silencing during persistent infection.

At the functional level, E5 is not only necessary for such repression, but also sufficient: its expression in uninfected keratinocytes markedly reduces IFN-κ levels, even in the presence of the E6 and E7 oncoproteins [29]. The mechanism by which E5 exerts this suppression involves multiple levels of interrelated molecular control. First, E5 alters EGFR dynamics, promoting its sustained recycling to the plasma membrane and generating continuous receptor activation. However, this signaling does not translate into IFN-κ activation; instead, it is redirected toward an immunosuppressive profile through a qualitative activation of the MEK/ERK pathway [6]. This repression is highly specific: E5 does not cause the general degradation of JAK/STAT pathway components or significantly alter global chromatin methylation. Instead, it exerts its effects through mechanisms that target the IFN-κ promoter and the functional capacity of key signaling pathways to induce its transcription [28].

**Figure 1:**
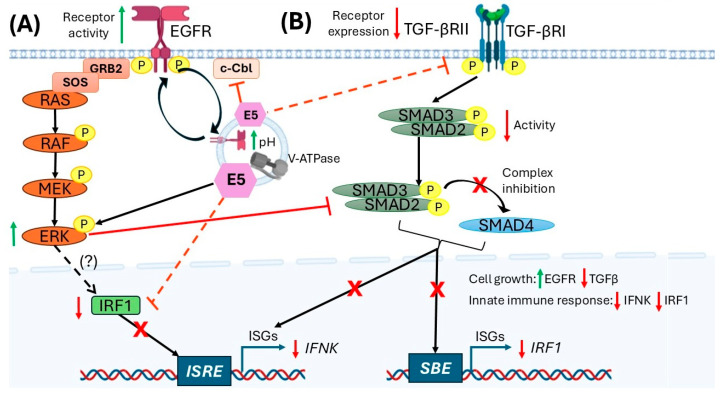
Mechanisms by which the HPV16 E5 oncoprotein regulates IFN-κ expression. The HPV16 E5 protein interferes with IFN-κ transcription by simultaneously modulating two key signaling pathways. (**A**) E5 alters EGFR trafficking and sustains MAPK/ERK activation, which indirectly reduces IRF1 transcriptional activity and limits its access to the IFN-κ promoter. (**B**) E5 decreases the expression of the TGF-β receptor II (TGF-βRII), thereby attenuating the phosphorylation and nuclear translocation of SMAD2/3/4 complexes and preventing the activation of SMAD-binding elements (SBE) within IFN-κ promoter. This dual repression of IRF1- and SMAD-dependent transcriptional mechanisms reduces IFN-κ mRNA levels and diminishes cytokine secretion, thereby evading the innate antiviral response during HPV16 infection. **X**, blocked pathway; “p” into yellow circle, means protein phosphorylation; red dashed line, indirect inhibition; red continuous line, direct inhibition; black continuous arrow, known positive signal; black dashed arrow, unknown positive signal; green up arrow, increase regulation; red down arrow, down regulation. (?) Unidentified signal pathway. Created with BioRender.com.

In this context, E5 interferes with the transcriptional function of IRF1, a key regulator of IFN-κ, without decreasing its protein levels. This interference was demonstrated in luciferase assays using a promoter containing an interferon-stimulated response element (ISRE), in which co-expression of E5 abolished IRF1-induced activation. Additionally, chromatin immunoprecipitation (ChIP) assays revealed a significant decrease in IRF1 occupancy at the IFN-κ promoter in the presence of E5 [6,30]. These findings suggest that E5 indirectly interferes with IRF’s transcriptional activity by blocking its interaction with the ISRE region in the ISG promoter, which reduces IFN-k expression. Together, E5 acts as a “functional interceptor” that selectively inactivates IRF1 at the IFN-κ promoter, blocking transcription of this antiviral cytokine and contributing to immune silencing in HPV16-infected epithelial cells [6] (Figure 1A).

Additionally, E5 interferes with the canonical TGF-β signaling pathway, one of the most potent inducers of IFN-κ in keratinocytes. Specifically, E5 reduces TGF-βRII receptor expression, limiting the cell’s ability to respond to TGF-β stimulation [31]. This repression is complemented by a decrease in the phosphorylation of SMAD3, the primary intracellular transducer of this pathway, which prevents its translocation to the nucleus and, consequently, the activation of regulatory elements such as the SMAD-binding element (SBE) in the IFN-κ promoter. This action blocks both SMAD-dependent signaling and TGF-β-mediated transcriptional activation, restricting another key pathway of IFN-κ induction [6] (Figure 1B). Together, these mechanisms allow E5 to strategically modulate distinct signaling pathways—EGFR, MAPK/ERK, IRF1, TGF-β/SMAD3, and JAK/STAT—to effectively silence the transcription of IFN-κ and antiviral effect genes, thereby contributing to evasion of innate immunity and to the episomal persistence of HPV16 in infected keratinocytes [6,24].

### 4.2. Inhibition of IFN’s Type I Expression

**Figure 2:**
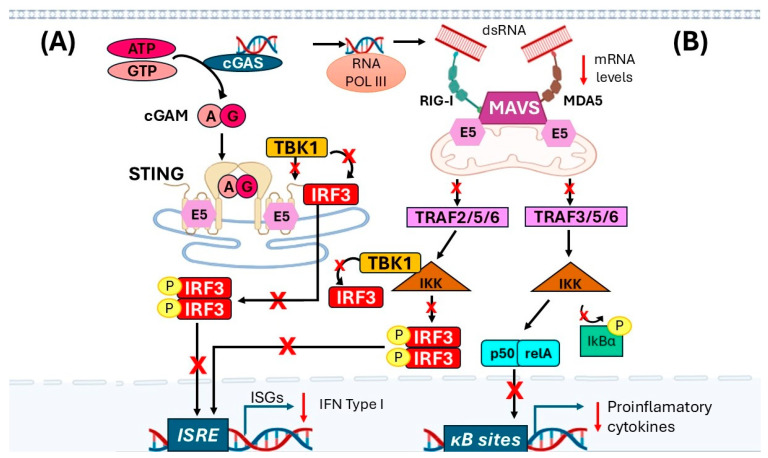
The role of E5 HPV in regulating the type I interferon response. (**A**) HPV16 E5 directly binds to both STING and MAVS, disrupting their downstream signaling. This interaction prevents IRF3 phosphorylation and nuclear translocation, thereby impairing IFN-β and ISG transcription. (**B**) Additionally, E5 directly interacts with MVAVS, disrupting the downstream cascade and reducing mRNA levels of RIG-I and MDA5, further blocking innate immune activation. Through this dual blockade, E5 effectively inhibits both cGAS–STING and RIG-I–MAVS pathways, contributing to viral immune evasion. **X**, blocked pathway; “p” into yellow circle, phosphorylated protein; black continuous arrow, known positive signal; red down arrow, downregulation. Created with BioRender.com.

Another critical signal activated by cytosolic viral DNA is the cyclic GMP-AMP synthase (cGAS), which, together with the stimulator of interferon genes (STING) (cGAS-STING), triggers the innate immune response in infected cells. The cGAS activated by viral DNA induces the synthesis of 2′3′-cyclic GMP-AMP (2′3′cGAMP) [32,33]. The 2’3’-cGAMP, which is a second messenger, activates STING, which is a transmembrane adapter protein found in the endoplasmic reticulum [34]. STING forms homodimers that bind to 2′3′-cGAMP and undergo conformational changes, allowing STING to move to the Golgi apparatus [35]. This complex then activates TANK-binding kinase 1 (TBK1) and interferon regulatory factor 3 (IRF3), a member of the IRF family that regulates the expression of interferon genes in response to viral infections. When IRF3 is phosphorylated, it dimerizes and translocates to the nucleus, inducing the expression of type-I IFNs [36] (Figure 2). It has been demonstrated that HPV16 E5 physically interacts with STING, as confirmed by co-immunoprecipitation assays in both overexpression systems and endogenously in HPV-positive cell lines. This direct binding of E5 to STING disrupts STING-mediated downstream signaling, thereby reducing IFR3 phosphorylation and nuclear translocation. Consequently, the transcriptional activation of type I IFN responses, including IFN-β1, and various ISGs, is significantly impaired [29] (Figure 2A). Through this mechanism, E5 effectively subverts the STING-dependent innate immune pathways, contributing to viral immune evasion and potentially influencing resistance to STING-targeted immunotherapeutic approaches.

As described in other viruses, AT-rich viral dsDNA accumulates in the cytoplasm and can be converted into RNA harboring a 5′ triphosphate moiety by RNA Pol III [37,38]. This allows cytosolic PRRs, such as retinoic acid-inducible gene I (RIG-I) and melanoma differentiation-associated protein 5 (MDA5), to recognize them and become activated [39,40].

The other antiviral signal that HPV modulates is MAVS (mitochondrial antiviral-signaling protein), which is the site of interaction between RIG-I and MDA5, and MAVS serves as an adaptor in signal transduction [41,42]. Downstream of this signaling pathway, TRAF interacts with the IκB complex (comprising IKKα, IKKβ, IKKε, and NEMO) and with nuclear factor κB (NF-κB) [43]. NF-κB is a heterodimer composed of p50/relA molecules, and when inactive, is maintained in the cytoplasm by its inhibitor IκB. Upon activation, it triggers a signaling cascade that degrades IκB, allowing NF-κB to translocate to the nucleus and increase the expression of inflammatory cytokines and type I IFNs [44,45]. During HPV infection, the E5 oncoprotein directly binds MAVS, thereby disrupting downstream MAVS-mediated signaling and impairing IRF3 phosphorylation and nuclear translocation [29,30] (Figure 2B). Consequently, E5 expression leads to significant suppression of IFN-β1 transcription and diminished expression of multiple ISGs, including RIG-I and MDA5, thereby attenuating the innate antiviral response [6,29].

Paradoxically, the studies suggested that, at late stages of infection, E5 could induce IFN-β production independently of the IRF-3/7 pathways, activating the alternative IRF-1-dependent pathway [30,46]. A possible explanation for this apparent contradiction in the role of E5 in downregulating IFN-β is that, at later stages of infection, the presence of IFN-β correlates with increased viral genome integration and, consequently, with the progression of high-grade lesions and cancer [6]. Additionally, it may contribute to a chronic pro-inflammatory environment that promotes the survival of tumor cells [47,48]. However, more studies are needed to support this finding.

Together, these findings reveal that E5 acts as a master regulator of antiviral gene expression, coordinating both upstream suppression of IFN-κ and downstream inhibition of ISG transcription, thereby creating a cellular environment conducive to viral persistence and immune evasion.

## 5. Role of E5 in Adaptive Immune Response Evasion

T-cells recognize antigens in the form of peptides derived from self or pathogen-derived proteins. T-helper (Th) cells (CD4+) recognize 14–25 aa-long peptides associated with MHC-II molecules. On the other hand, cytotoxic T (Tc) cells (CD8+) recognize 8–10 aa-long peptides associated with MHC-I molecules. Upon antigen recognition, T-cells are activated and differentiate into effector cells. T cells secrete cytokines that promote B and Tc cell proliferation and differentiation, and Tc cells recognize and eliminate cancer cells and cells infected with intracellular pathogens [49,50].

### 5.1. Altered CD4+ T Cell Activation

Antigen-presenting cells use complex machinery to process and display peptide-MHC complexes on their cell surface for recognition by T cells. MHC-II consists of an α and β chain that assemble in the ER and are stabilized by the invariant chain (li) [51]. The MHC-II-li complex is transported from the Golgi apparatus to a compartment called the MHC class II compartment (MIIC) [51]. At acidic pH, the proteases cathepsin S and L are activated and digest li, generating a residual Class II-associated invariant chain peptide (CLIP) in the MHC-II binding zone of the molecule [52]. Subsequently, an exchange occurs between CLIP and an antigenic peptide derived from proteins degraded in the endocytic pathway. MHC-II molecules bearing the peptide are transported to the cell plasma membrane, where they can interact with CD4+ T cells [53].

**Figure 3:**
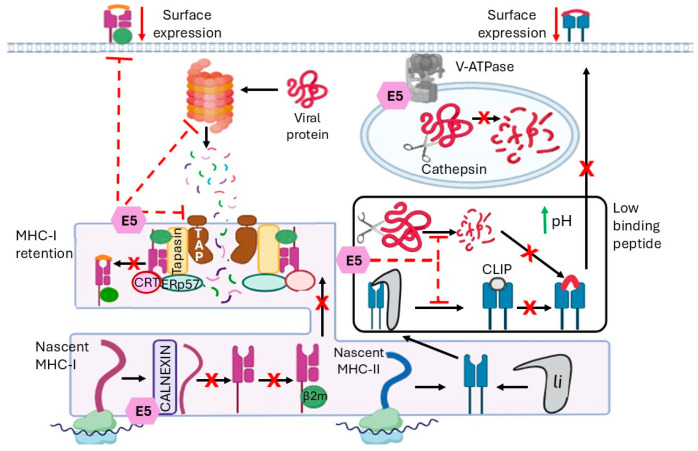
Regulation of antigen processing and presentation to T cells by the HPV16 E5 protein. The E5 oncoprotein interferes with the MHC-I and MHC-II pathways. (Left Panel)**:** The MHC-I Pathway. In the ER, E5 interacts with the MHC-I heavy chain and the chaperone protein Calnexin, thereby sequestering the MHC-I complex within the Golgi apparatus and preventing its translocation to the plasma membrane. The absence of viral peptide presentation to CD8+ T cells allows the infected cell to evade recognition by cytotoxic T lymphocytes. (Right Panel): The MHC-II Pathway. E5 disrupts the maturation of MHC-II molecules by inhibiting the V-H+-ATPase proton pump, thereby increasing endosomal pH and inhibiting the activity of proteases (Cathepsins), which are essential for degrading the Invariant Chain (Ii), which prevents the formation of the CLIP fragment and the loading of exogenous viral antigens. Additionally, E5 reduces MHC-II surface expression, thereby preventing the activation of CD4+ T cells and a robust immune response. **X**, blocked pathway; black continuous arrow, known positive signal; green up arrow, increase in pH; red down arrow, downregulation; red dashed line, indirect inhibition. Created with BioRender.com.

In this specific antigen presentation pathway, HPV16 E5 inhibits MHC-II maturation by blocking endosomal processing of the invariant MHC-II chain, thereby preventing peptide loading and transport of MHC-II to the cell surface [54]. It achieves this by interacting with the 16 kDa C subunit of the V-H+ATPase and reducing its ability to acidify endocytic compartments [54] (Figure 3, right panel).

### 5.2. Altered CD8+ T Cell Activation

MHC-I is translated and assembled in the ER through the chaperone calnexin, which stabilizes the heavy chain before it associates with β2-microglobulin [55]. Before peptide binding, MHC-I is bound to chaperone proteins such as calreticulin, Erp57, and tapasin, forming the peptide-loading complex (PLC) [55,56]. Tapasin interacts with the transporter associated with antigen processing (TAP), which translocates peptides from the cytoplasm to the ER [56], and these peptides bind to the heavy chain of the MHC molecule. TAP is a heterodimeric protein, composed of TAP1 and TAP2 subunits that function as an ATPase pump for the peptides generated in the cytosol from cytosolic proteins degraded by the proteosome enzymatic complex [57]. This transporter carries peptides of 8 to 16 amino acids, which undergo further processing before binding to MHC-I molecules. Once the MHC-I molecule is assembled with β-2 microglobulin and the peptide, the complex is transported through the Golgi apparatus to the cell membrane, where it is recognized by Tc-cells [58] (Figure 3, left panel).

To evade the immune system during HPV infection, E5 can block MHC-I transport, leading to a decrease in MHC-I-peptide complexes on the keratinocyte surface, diminishing recognition of the viral antigen and reducing activation of Tc cells [28,59]. The di-leucine motifs within the N-terminal hydrophobic transmembrane domain of HPV16 E5 mediate the retention of MHC-I heavy chains in the ER/Golgi by associating with the chaperone calnexin; this retention specifically prevents properly folded MHC-I from reaching the cell surface, reducing antigen presentation and promoting immune evasion, an effect absent in calnexin-deficient cells [28,60,61] (Figure 3, left panel).

The immunoproteasome is an isoform of the proteasome that processes intracellular antigens for presentation by MHC-I. Stimulation with IFN-γ induces exchange of the proteasome subunits β1, β2, and β5 for their corresponding immune subunits β1i (PSMB9, LMP2), β2i (PSMB10, LMP10), and β5i (PSMB8, LMP7) [62,63]. This change increases the activity of chymotrypsin and trypsin, resulting in a greater number of peptides available for binding to MHC-I molecules [64,65]. Another function is to facilitate the degradation of poly-ubiquitinated proteins, prevent the accumulation of misfolded and misassembled proteins, and maintain cell viability [66]. However, in tumor cells, immunoproteasome inhibitors have been reported to be used to their advantage, thus avoiding cell death [67,68].

In this part of the system, the presence of E5 leads to a significant reduction in the expression of PSMB8/Psmb8 (encoding LMP7) and PSMB9/Psmb9 (encoding LMP2) mRNA and protein [29]. This results in reduced proteolytic activity required for peptide cleavage and a reduced repertoire of peptides available for loading onto MHC-I. Furthermore, Miyauchi and colleagues reported that, in Head and Neck Squamous Cell Carcinoma (HNSCC) patients, low levels of E2/E4/E5, PSMB8, and PSMB9 expression were associated with lower disease-free survival and overall survival than in those with high levels [29].

Finally, TAP is regulated by the STAT1 pathway and shares a bidirectional promoter with the PSMB9 gene. As mentioned above, E5 negatively regulates these genes, thereby reducing peptide transport from the cytosol to the ER and, consequently, MHC-I-peptide complexes [29].

## 6. Prophylactic and Therapeutic E5-Based Vaccine Against HPV-Induced Tumors

Therapeutic strategies against HPV have increasingly considered the E5 oncoprotein a promising immunological target due to its involvement in cell-cycle regulation, modulation of immune responses, and early expression during cervical carcinogenesis. Despite its potential, E5 remains untargeted by ongoing immunotherapy protocols for early HPV-induced lesions or tumors. To date, only preclinical evidence exists from E5-targeted immunotherapies. One primary strategy utilized a bioinformatically selected Tc cell epitope (LSVSTYTSL, aa 29–37) formulated with CpG-ODN 1826 adjuvant. When evaluated in C57BL/6 mice, this vaccine was highly effective since successfully prevented tumor development following rTC-1 cell challenge (E5/E6/E7+) and induced a robust IFN-γ and IL-2-producing T cell response. Furthermore, in the therapeutic model, vaccination significantly reduced established tumor volumes and extended survival. This immune activity was strictly antigen-specific, as no antitumor effects were observed in E5-negative control models [69].

Liu et al. (2000) evaluated a recombinant adenoviral vector (rAd) encoding the E5 protein in C57BL/6 mice [70]. Both prophylactic and therapeutic immunization with rAd-E5 significantly delayed the growth of TC-1/E5/E6/E7 tumor cells for up to three weeks post-challenge. This protective effect was mediated by CD8+ T cells, confirming that E5 can trigger robust cytotoxic activity. This evidence further validates the potential of E5 as a key immunological component in therapeutic vaccination strategies aimed at controlling HPV-induced cancers [70].

Alternatively, the second-generation DNA vectors pVAX-E5CP and pVAX-E5MultiCP, encoding the full-length E5 protein and a set of immunogenic epitopes, respectively, were tested in C57BL/6 mice. Both vectors were designed as fusions with the Potato virus X (PVX) capsid protein (CP) to enhance immunogenicity. In mice vaccinated with a four-dose regimen, pVAX-E5CP elicited a robust CD4+ T cell response that surpassed that induced by similar anti-E6 or anti-E7 vaccines. Notably, it induced four times higher IFN-γ secretion than pVAX-E7CP. Furthermore, in a tumor model induced by C3-Luc cells (expressing E5, E6, and E7), 80% of mice vaccinated with pVAX-E5CP remained tumor-free, compared with 40% of mice vaccinated with pVAX-E5MultiCP or pVAX-E7CP, with significantly lower tumor burdens. This antitumor effect was closely associated with the activation of E5-specific CD4+ T cells, which are critical for generating immunological memory and providing help to CD8+ T effector cells [71].

Finally, a strategy targeting the DEC-205 molecule on dendritic cells was evaluated by conjugating full-length E5 protein to an anti-DEC-205 monoclonal antibody. In the BMK-16/C-MYC cell (expressing E5/E6/E7) model in BALB/c mice, therapeutic administration of this conjugate elicited a strong protective immune response, with 70% of animals achieving tumor control and survival, while the remaining 30% succumbed by day 72. This vaccine induced E5-specific protective CD4+ and CD8+ T cells with a Th1/Th17-type cytokine profile. Further, treatment of vaccinated mice with anti-PD-1 monoclonal antibody delayed tumor growth for an extra 20 days [72].

Taken together, these studies converge on the conclusion that the E5 oncoprotein represents a functional and clinically relevant immunological target. Its inclusion across various vaccine platforms—ranging from peptide-based and DNA vaccines to viral vectors and antibody-based delivery systems—has consistently demonstrated the capacity to reduce tumor burden and, in many cases, to achieve complete tumor regression in preclinical models. These findings reinforce the clinical relevance of targeting E5, particularly during early-stage infection and lesion progression, where E5 expression is most prominent and accessible to immune recognition.

## 7. Conclusions and Future Directions

All 14 HR-HPV types share the same basic genomics blueprints. E6 and E7 are highly conserved across HR-HPV types and maintain their core functions: binding and degrading tumor suppressor proteins (p53 and pRb, respectively). Meanwhile, E5 is the most variable among the HR-HPV types [73]. For instance, while HPV16 E5 is highly hydrophobic with three transmembrane domains, other types can vary in length and membrane orientation, and expression can be lost during viral genome integration into host DNA once the cell is fully transformed [8]. However, persistent expression of E6 and E7 is required to maintain the malignant phenotype of cancer cells. In contrast, in LR-HPVs, the viral DNA typically remains episomal, maintaining E5 within its natural regulatory framework and contributing to benign proliferation without inducing the genomic instability characteristic of HR-HPV integration [59]. Therefore, the oncogenic potential of the viral type results from synergy between the combined efficacy of E6/E7 in dismantling p53/pRb, which is essential for cancer maintenance, and the supportive activity of their specific E5, which enhances the early stages of transformation by promoting cell proliferation and immune evasion [74,75].

Immune response evasion is a central mechanism by which HPV establishes persistent infections and promotes tumor development in susceptible epithelial tissues. From the initial stages, the viral cycle is associated with the stratified squamous epithelium, preventing access by immunocompetent cells and the generation of inflammatory signals [76]. This localization, along with the absence of viremia, necrosis, or cell lysis, prevents activation of danger receptors and delays viral recognition, allowing a state of functional latency in which antigenic exposure is minimal, and the virus persists for a prolonged period without inducing an effective immune response [77].

The viral oncoproteins E5, E6 and E7 coordinately modulate innate and adaptive immunity: E5 and, to a lesser extent, E6 reduce MHC-I expression, limiting antigen presentation and recognition by CD8^+^ T lymphocytes [78], while all of them alter type I IFN signaling and the cGAS/STING pathway, suppressing the early antiviral response and favoring an immunologically silent environment [29,78]. At the adaptive level, HPV shifts T lymphocyte polarization from a cytotoxic Th1 profile to a Th2/regulatory one (IL-4, IL-10, IL-13), reducing cytotoxic efficacy and promoting local tolerance [79]. Persistent infection facilitates viral DNA integration and sustained expression of E6 and E7, leading to genomic instability, cell cycle disruption, and escape from immunosurveillance, critical steps in neoplastic transformation of the epithelium [2,3].

Overall, HPV immune evasion is a multifactorial and dynamic process that compromises viral sensing, antigen presentation, and interferon-mediated responses. This process not only allows viral persistence but also establishes an immunosuppressive microenvironment that facilitates tumor progression [80]. Therefore, understanding the immunomodulatory mechanisms of HPV is essential for designing more effective preventive and therapeutic strategies. Unlike E6 and E7, whose expression is associated with more advanced stages of transformation, E5 is expressed early and actively contributes to immune evasion by modulating antigen presentation and interferon signaling [28,29,71]. Consequently, the same mechanisms that allow E5 to orchestrate immune subversion also render it a promising target for immunotherapy. Thus, incorporating E5 into peptide, DNA, or recombinant viral vector platforms, researchers have elicited robust CD4+/CD8+ T cell immunity and a Th1-biased response, leading to potent anti-tumor activity [69,70,71,72,81]. Consequently, the incorporation of E5 into multi-epitope therapeutic vaccines and its combination with immune checkpoint inhibitors (anti-PD-1, anti-CTLA-4) could enhance antitumor immunity and improve clinical outcomes in HPV-induced cancers.

## Data Availability

No new data were created in this review.

## Abbreviations

The following abbreviations are used in this manuscript:

cGAS	Cyclic GMP-AMP synthase
2′3′cGAMP	2′3′-cyclic GMP-AMP
CC	Cervical Cancer
CIN	Cervical Intraepithelial Neoplasia
CLIP	Class II-Associated Invariant Chain Peptide
COX-2	Cyclooxygenase-2
EGFR	Epidermal Growth Factor Receptor
HNSCC	Head and Neck Squamous Cell Carcinoma
HR-HPV	High-risk Human Papillomavirus
IFNs	Interferons
IRAK1	Interleukin-1 receptor-associated kinase
IRF	Interferon Regulatory Factor
ISGs	Interferon-stimulated genes
MAPK	Mitogen-Activated Protein Kinase
MAVS	Mitochondrial antiviral-signaling protein
MDA5	Melanoma differentiation-associated protein 5
MHC	Major Histocompatibility Complex
MIIC	MHC class II compartment
NF-κB	Nuclear factor-κB
PGE _2_	Prostaglandin E2
PKA	Protein kinase A
PRRs	Pattern recognition receptors
RIG-I	Retinoic acid-inducible gene I
SOCS3	Suppressor of Cytokine Signaling 3
STAT1	Signal Transducer and Activator of Transcription 1
STING	Stimulator of interferon genes
TGF-βR	Transforming growth factor beta receptor
TAP	Transporter Associated with Antigen Processing
TBK1	TANK-binding kinase 1
TRAF	TNF receptor-associated factor
TRAIL	TNF-related apoptosis-inducing ligand
